# *Eucalyptus camaldulensis* Extract Mediated Green Synthesis of Iron Oxide Nanoparticles and In Vitro and In Vivo Biological Screening, and Detailed Molecular Docking Analysis

**DOI:** 10.1002/fsn3.71992

**Published:** 2026-06-15

**Authors:** Abdur Rauf, Arshad Iqbal, Shahkar Usama, Imran Ullah, Walaa F. Alsanie, Abdulhakeem S. Alamri, Zuneera Akram, Rahaf Ajaj, Naveed Muhammad, Zubair Ahmad, Jaseem Ahmad, Mohammed Mansour Quradha

**Affiliations:** ^1^ Department of Chemistry University of Swabi Anbar Khyber Pakhtunkhwa Pakistan; ^2^ Department of Crystallography and Structural Physics, Institute of Condensed Matter Physics Friedrich‐Alexander University Erlangen Germany; ^3^ Department of Clinical Laboratory Sciences, The Faculty of Applied Medical Sciences Taif University Taif Saudi Arabia; ^4^ Research Center for Health Sciences Taif University Taif Saudi Arabia; ^5^ Department of Pharmacology, Faculty of Pharmaceutical Sciences Baqai Medical University Karachi Pakistan; ^6^ College of Engineering Abu Dhabi University Abu Dhabi UAE; ^7^ Department of Pharmacy Abdul Wali Khan University Mardan KPK Pakistan; ^8^ Seiyun University College of Education Seiyun Hadhramawt Yemen; ^9^ Pharmacy Department, Medical Sciences Aljanad University for Science and Technology Taiz Yemen

**Keywords:** biological activities, *Eucalyptus camaldulensis*, extracts, IONPs, molecular docking analysis, phytochemicals screening

## Abstract

Natural products and their secondary metabolites have long been known as good bioresources in the synthesis of nanomaterials. In this paper, IONPs were synthesized by an eco‐friendly method using the aqueous extract of *Eucalyptus camaldulensis* and evaluated for their physicochemical properties, enzyme inhibitory activity, pharmacological potential, and molecular docking. The synthesized IONPs were subsequently characterized by UV–Vis, FTIR, and SEM techniques to determine the functional groups and surface morphology. In addition to that, the synthesized IONPs were also tested in terms of in vitro (urease, carbonic anhydrase II, and xanthine oxidase inhibition) and in vivo (analgesic and sedative) activity. The prepared IONPs demonstrate effective enzyme inhibitory activity (95.49% urease, 72.23% CA‐II, 86.03% XO) and dose‐dependent sedative and anti‐writhing effects. However, direct comparison with the crude extract on a mg/kg basis is limited by differences in phytochemical content between the two formulations. Quercetin, a flavonoid previously reported in *E. camaldulensis* , was used as a representative ligand for molecular docking, while a simplified iron‐based cluster model was used to explore possible interactions of the nanoparticle surface with target enzymes. Thus, these results suggest that *E. camaldulensis* ‐mediated IONPs possess promising enzyme inhibitory and sedative properties, but further physicochemical, kinetic, mechanistic, toxicity, and biophysical validation studies are required.

## Graphical abstract

*Eucalyptus camaldulensis* extract mediated green synthesis of IONPs and In vitro and In vivo Biological Screening, and Detailed Molecular Docking Analysis.

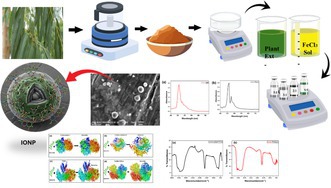

## 1 Introduction

Nanotechnology involves the design, synthesis, and manipulation of materials at the atomic, molecular, or nanoscale level to obtain unique physicochemical properties (Saif et al. 2016). Nanostructures are typically defined as structures with sizes that are between 1 and 100 nm in size and have a high surface area to volume ratio, giving properties that find great utility in mechanics, optics, and electronics through biology and medicine (Din, Ajaj, et al. 2025; Herlekar et al. 2014; Rauf, Ibrahim, et al. 2025; Ullah, Neder, Irfan Khan, et al. 2025; Ullah, Neder, Parwaz, et al. 2025; Ullah, Neder, Sufian, et al. 2025; Yang et al. 2025). However, one of the major disadvantages of conventional methods for synthesizing nanoparticles is related to the utilization of toxic and heavy reducing agents such as sodium borohydride and hydrazine hydrate, which are very reactive toward many hazardous/toxic waste materials that may cause irreversible harm to both human life and the environment (Devatha et al. 2016). Plant‐mediated synthesis is a green approach in which phytochemicals present in plant extracts can assist in the reduction, nucleation, stabilization, and capping of metal or metal oxide nanoparticles (Gottimukkala et al. 2017). Green synthesis is preferred over the conventional chemical and physical methods due to its low cost, eco‐friendly nature, and extensibility for a larger scale of production without high pressure, temperature, or hazardous chemicals (Huang et al. 2014). Green strategies or approaches have been developed to synthesize nanoparticles: the bottom‐up approach, where atoms and molecules are assembled to produce nanomaterials with well‐defined size and shape; and top‐down synthesis also provides improved crystal growth and stability (Kaur and Chopra 2018; Ullah et al. 2024).

Nanomaterials have received much attention as prospective candidates, and iron nanoparticles (Fe NPs) are one class of these nanomaterials, in which their size makes them unique from bulk materials due to their unusual physical, chemical, and magnetic properties. These nanoparticles, which generally measure between 1 and 100 nm, possess a high surface‐to‐volume ratio, translating into high reactivity (e.g., catalytic efficiency and adsorption capacity) compared with their bulk counterparts (Din, Ali, et al. 2025; Rauf, Ibrahim, et al. 2025; Romero‐Cedillo et al. 2020). Various applications ranging from magnetic and electrical applications to biomedical ones, such as biological labeling, biomagnetic separation, target drug delivery, systemic therapy, wherein fiber suspension of Iron is used, which shortens spinning relay time, causing local temperature increase, also suitable for hyperthermia treatment (Huber 2005).

Iron nanoparticles as a conjugate have the dual functions of serving as both an MRI contrast agent and drug carrier since they are used in administering controlled drug techniques targeting cancer treatment and diagnosis (Zhang and Elliott 2006). One of the primary applications for intentional iron oxide nanoparticles is molecular imaging. The predominant use of targeted iron oxide nanoparticles has been for in vitro and animal work: only a few focus on the clinical application of iron oxide nanoparticles (Bhuiyan et al. 2020). Along with the negatively charged magnetic particle surface, a powerful way to load enough micromolar concentration of Fe in cultured cells by using either cationic transfection agents or cell‐permeable peptide initially provides an innovative technique for MRI‐based labeling and tracking cells in a living system (Góral et al. 2023).

The main use case of iron nanoparticles is environmental remediation, and it can help to eliminate or, by imitation, reduce heavy metals, synthetic poisons, as well as other pollutants from different water bodies and soils. The high surface area and large reactivity of IONPs can be regarded as promising adsorbent/reducing agents for converting or degrading harmful compounds (Ebrahiminezhad et al. 2018).

In the field of catalysis, iron nanoparticles have been explored for their role in various chemical processes, including the production of hydrogen, the conversion of greenhouse gases, and the synthesis of valuable chemicals (Chala 2023). Their tunable catalytic activity and high surface area make them attractive candidates for these applications (Montiel Schneider et al. 2022). There are various chemical methods for preparing iron oxide nanoparticles, one of which is the hydrothermal precipitation method (Devatha et al. 2016). This approach has garnered significant interest because of its benefits, including rapid reaction times, excellent control over particle morphology, and minimal impurities in the particles. Another widely utilized method for synthesizing magnetic nanoparticles is chemical coprecipitation (Kandpal et al. 2014).

*Eucalyptus camaldulensis* forests represent the largest areas of both cultivated and natural hardwood species globally, belonging to the Myrtaceous family. The *Eucalyptus* genus includes over 700 species found worldwide, with significant plantations in countries such as Brazil, Australia, Egypt, China, Portugal, the European Union, and Spain (Devatha et al. 2016). Generally, Eucalyptus species are perennial and evergreen, ranging from small shrubs to towering trees. *Eucalyptus* leaves display shape dimorphism between juvenile and mature stages: juvenile leaves are nearly oval, sessile, and glaucous sometimes, while mature leaves are strong, oval, and oblong. The bark of Eucalyptus varies widely, including permanent, deciduous, coarse, and soft varieties. Bark characteristics like the length of the fiber, texture, color, and stiffness vary with the age of the plant (Gour and Jain 2019). The woody fruits of *Eucalyptus* come in various shapes, including globular, conical, and hemispherical, while their seeds range in size from 1 mm to 2 cm and can be spherical or elliptical. *Eucalyptus* species are rich in bioactive compounds with antimicrobial properties, such as flavonoids, tannins, phenolics, phloroglucinols, terpenoids, and cardiac glycosides. Although Eucalyptus bark is often discarded as waste, it serves as an excellent fuel source (Harikrishnan et al. 2023). The bark of species like *Eucalyptus grandis* and *Eucalyptus citriodora* is high in tannins, which are essential for producing wood adhesives. Eucalyptus bark demonstrates significant sorption efficiency, making it effective in treating effluents contaminated with chromium (Cr). Studies indicate that Eucalyptus bark and its derivatives can be utilized to remediate polluted aqueous solutions.

*E. camaldulensis* belongs to the kingdom *Plantae*, phylum *Tracheophyte*, class *Magnoliopsida*, order *Myrtales*, family *Myrtaceae*, genus *Eucalyptus*, and its specific epithet is *Camaldulensis* (Berman et al. 2019; Harikrishnan et al. 2023).

Various *Eucalyptus* species have long been utilized in traditional medicine for their antiseptic properties and efficacy against sinus congestion, influenza, and common colds, which are upper respiratory tract infections (Ashraf et al. 2015). The essential oil that these plants yield is commonly used for treating pulmonary infections through inhalation (Sahin Basak and Candan 2010). Prior research on the essential oil from *E. camaldulensis* flowers has identified 1,8‐cineole, β‐pinene, and spathulenol as the main components. The leaves' essential oil is notable for its high levels of p‐cymene, γ‐terpinene, α‐pinene, 1,8‐cineole, terpinen‐4‐ol, α‐terpineol, carvacrol, and thymol (Chiamaka et al. 2019). The fruits' essential oil is abundant in aromadendrene, α‐pinene, drimenol, and cubenol (Singab et al. 2011). Additionally, studies have isolated calmodulin, ursolic acid lactone acetate, and Ursolic acid lactone as pentacyclic triterpenoids from *E. camaldulensis* , all of which demonstrate spasmolytic activity (Chiamaka et al. 2019). Subsequently, eucalyptanoic acid, another triterpenoid acid, was also isolated and exhibited spasmolytic properties (Ashraf et al. 2015). Flavonoid glycosides have been extracted from *E. camaldulensis* leaves. Due to their inherent properties, *Eucalyptus* species are potential sources of antimicrobial compounds, crucial for their defense mechanisms against pathogens (Ghareeb et al. 2018; Huang et al. 2015).

Although research on green synthesis is expanding, limited studies have connected *E. camaldulensis* ‐mediated IONPs with combined physicochemical characterization, in vitro enzyme inhibition, in vivo pharmacological evaluation, and computational docking. Therefore, the present study aimed to synthesize IONPs using *E. camaldulensis* leaf extract, characterize the prepared material, evaluate enzyme inhibitory and in vivo biological effects, and use molecular docking as a hypothesis‐generating approach to explore possible interactions with selected enzyme targets.

## 2 Materials and Methods

### 2.1 Reagents and Chemicals

Ferric chloride hexahydrate (FeCl_3_·6H_2_O), methanol, ethanol, n‐hexane, ethyl acetate, sodium hydroxide (NaOH), dimethyl sulfoxide (DMSO), and distilled water were used during extraction, phytochemical screening, nanoparticle synthesis, and biological assays. All reagents were of analytical grade.

### 2.2 Equipment and Apparatus

The instruments used in the preparation of IONPs include an analytical balance for precise measurement of reagents, a UV–Visible Spectrophotometer (Shimadzu UV‐2600) for monitoring the absorbance, confirming nanoparticle synthesis, and a magnetic stirrer for uniform mixing of reactants. Glassware such as beakers, flasks, and pipettes are used for handling and mixing solutions. A pH meter was used for pH monitoring, while standard laboratory glassware was used for solution preparation and handling.

### 2.3 Preparation of the Plant Extract

*E. camaldulensis* leaves were collected from the district of Swabi, Khyber Pakhtunkhwa, Pakistan and were thoroughly washed with double distilled water to remove the surface contaminants and were then dried at ambient temperature ranging 25°C–28°C, under shaded conditions, with relative humidity ranging from 45% to 55% for a period of 14 days until a constant weight was achieved. The dried leaves were then ground into a fine powder by grinding the dried leaves using a mechanical grinder (Bhuiyan et al. 2020). Four grams of the powdered plant material was precisely weighed and macerated in 400 mL of distilled water for 48 h for extract preparation, with periodic stirring at regular intervals to ensure efficient extraction. The resulting mixture was filtered three times through Whatman No. 1 filter paper to obtain a clear, pure extract. This extract (aqueous) was then used for the green synthesis of iron nanoparticles, while a working solution of 1 mM FeCl_3_·6H_2_O was prepared separately in distilled water (Gottimukkala et al. 2017).

### 2.4 Synthesis of IONPs

To synthesize IONPS various ratios of the prepared plant extract and the 1 mM FeCl_3_·6H_2_O working solution were mixed to create different preparations (1:1, 2:1, 3:1, 5:1, and 9:1). Each mixture was then transferred into clean vials and stirred for 24 h continuously at room temperature by using a magnetic stirrer. The color change from yellow to black during this process indicates the successful formation of iron Nanoparticles.

### 2.5 Characterization of IONPs

To confirm the synthesis of IONPs, various analyses were performed to evaluate optical properties, functional groups, and morphology. The solutions were analyzed using a UV‐Visible spectrophotometer (Rauf, Ahmad, et al. 2025). According to the spectrophotometer results, the optimal ratio for the synthesis of IONPs was found to be 3:1 in *E. camaldulensis* plant extract. To determine the functional groups responsible for iron nanoparticle production, a Fourier transform infrared (FTIR) spectrophotometer was used. The FTIR spectrum was recorded between 4000 and 400 cm^−1^. For the confirmation of the morphology of these nanoparticles, scanning electron microscopy (SEM) was utilized to develop an image of the sample. The dried samples are secured to a sample holder with double‐sided conductive tape. This ensures proper conductivity and prevents sample movement during imaging. The samples are then coated with a thin layer of platinum–gold to enhance their conductivity and improve image quality by reducing charging effects. Once prepared, the samples are placed in the microscope, and at an operating voltage of 80 kV, the structural characteristics of the IONPs (iron nanoparticles) are analyzed (Herlekar et al. 2014).

### 2.6 Phytochemical Analysis

For phytochemical screening, different solvent extracts of *E. camaldulensis* leaves were prepared using n‐hexane, ethyl acetate, methanol, and distilled water.

#### 2.6.1 Alkaloid

The 0.5 g of crude extract from each plant was boiled with 2% H_2_SO_4_ and then mixed with Dragendorff reagent. The presence of alkaloids was revealed by an orange‐red precipitate (Huang et al. 2014).

#### 2.6.2 Tannins

Small amounts of each crude extract (0.2 g) were boiled and filtered. To the filtrate, ferric chloride was added dropwise. A dark green coloration indicated the presence of tannins (Ijaz et al. 2020).

#### 2.6.3 Flavonoids

Mix a small amount of the plant's crude extract with the AlCl_3_ solution. Observe for the development of a yellow color, indicating the presence of flavonoids (Kharissova et al. 2013).

#### 2.6.4 Carbohydrates

Add a few drops of α‐naphthol reagent to the plant's crude extract. Carefully layer concentrated sulfuric acid down the side of the test tube to form a lower layer (Shahwan et al. 2011). A purple or violet ring at the interface of the two layers indicates the presence of carbohydrates (Kharissova et al. 2013).

#### 2.6.5 Saponins

Vigorously shake the crude plant extract with distilled water in a test tube. If persistent froth forms and lasts for at least 15 min, this suggests the presence of saponins (Kuang et al. 2013).

#### 2.6.6 Anthraquinones

Extract the plant's crude with chloroform or diethyl ether. Separate the organic layer and add dilute ammonia solution to it. A pink, red, or violet color in the ammoniacal layer indicates the presence of anthraquinones.

### 2.7 In Vitro Enzymes Inhibitions

Enzyme inhibition assays, urease, carbonic anhydrase II (CA II), and xanthine oxidase (XO) were systematically studied on the biological activities of the synthesized IONPs in accordance with the standard protocols.

#### 2.7.1 Urease Inhibition

The urease inhibitory potential of the synthesized IONPs and E. camaldulensis extract was assessed against jack bean urease by using a microplate‐based protocol. Each reaction well contained 25 μL of urease solution, 55 μL of urea buffer (100 mM), and 5 μL of the test sample, followed by incubation at 30°C for 15 min. The ammonia generated through enzymatic hydrolysis was quantified using the indophenol method (Ojha et al. 2026). To each well, 45 μL of phenol reagent (comprising 1% w/v phenol and 0.005% w/v sodium nitroprusside) and 70 μL of alkali reagent (0.5% w/v NaOH with 0.1% active chlorine from NaOCl) were sequentially added. After an additional 50‐minute incubation period, absorbance readings were recorded at 630 nm using a microplate reader. With the total assay volume maintained at 200 μL, all experiments were conducted in triplicate. Thiourea served as the reference standard, and the following equation was used to calculate percent inhibition (Ullah et al. 2024).

$$\text{Percent effect}=100-\frac{{\mathrm{OD}}_{\text{testwell}}}{{\mathrm{OD}}_{\text{control}}}\times 100$$

#### 2.7.2 CA II

To evaluate the CA II inhibitory activity of the synthesized IONPs, CO_2_ hydration was monitored using a pH‐indicator‐based method. The assay mixture—comprising the IONPs, CA II enzyme, and substrate—was prepared under optimal temperature and pH conditions to preserve enzymatic activity. Following incubation, pH variation was detected using an appropriate indicator dye (Ekinci et al. 2013). Percent inhibition was then calculated by comparing the pH changes in the test samples against control wells containing the enzyme without an inhibitor. To confirm data reliability and reproducibility, all experiments were performed in triplicate (Kandpal et al. 2014).

#### 2.7.3 XO

The XO inhibitory property of the synthesized IONPs was identified by observing the hydroxylation of xanthine to uric acid, which has an absorbance at 295–296 nm. The assay mixture was reconstituted with the test sample (IONPs dispersed in DMSO), phosphate buffer, XO enzyme, and xanthine as the substrate. Namely, 10 μL of the solution in the test sample was combined with 20 μL of phosphate buffer (including 0.003 units of XO) and 20 μL of xanthine (0.1 mmol/L). It reacted with the addition of XO, and the incubation period was 10 min at room temperature. The formation of uric acid was measured by registering an absorbance at 295 nm on a microplate reader, and the readings were taken at 1‐min intervals up to 15 min. The level of XO activity inhibition was determined by the treatment and control results, which were compared in terms of absorbance. The values of IC_50_ were estimated in EZ‐Fit software. Allopurinol served as a reference standard, and all experiments were replicated three times to make them reproducible (Kandpal et al. 2014).

#### 2.7.4 Sample Preparation for Enzyme Assays

To achieve a stock concentration of 1 mg/mL, all test samples (crude extract and IONPs) were dissolved in DMSO. For enzyme inhibition assays, serial dilutions were prepared to reach final concentrations ranging from 0.1 to 1000 μg/mL. Immediately prior to each assay, IONP suspensions were dispersed using an ultrasonication bath (Elmasonic S30, 37 kHz) for 15 min to prevent aggregation and ensure uniform particle dispersion. Vehicle controls which contained an equivalent volume of DMSO (final concentration ≤ 1%) were included in all experiments (Marslin et al. 2018), where no significant interference with enzyme activity was observed.

### 2.8 In Vivo Study

BALB/c mice of either sex with an average weight of 22–25 g were purchased from the National Institute of Health (NIH), Islamabad, Pakistan. The animals were shifted to the animal house of the Department of Pharmacy, Abdul Wali Khan University, Mardan. The standard temperature was maintained during the transportation of animals. A good, recommended laboratory condition was provided to the animals with a temperature of 25°C, and standard food was fed with fresh water and libitum. Animals were classified as negative control, positive control, and groups to be tested. Each group consisted of eight animals. A special marker was used to assign the number of animals in each group. Only the healthy animals were included in the experimental procedure. Any animal with unusual vocalization or hindered movement, and so forth, was not included in the experiment.

Before the experimental procedures, the study was approved by the ethical committee of the Department of Pharmacy, Abdul Wali Khan University, Mardan with ethical committee No. AWKUM/Pharm 889. According to the International Animals' Protection Act/Ethical Act, each of the animals after the experiment must be disposed of. So, all the used animals were properly disposed of by cervical dislocation.

#### 2.8.1 Analgesic Activity: (Acetic Acid‐Induced Writhing)

The analgesic evaluation of the selected samples was carried out through the acetic acid‐induced writhing model. The animals in this model were divided into groups (*n* = 8 per group): negative control (normal saline, 10 mL/kg, IP), positive control (diclofenac sodium, 10 mg/kg, IP), and test groups receiving extract (25, 50, and 100 mg/kg, IP) or iron nanoparticles (2.5, 5, and 7.5 mg/kg, IP). Thirty minutes posttreatment, acetic acid (0.6% v/v, 10 mL/kg, IP) was administered to all animals (Mamun‐Or‐Rashid et al. 2017); the experimental design did not include a control group treated with IONPs alone (without the acetic acid challenge). This omission limits the ability to determine whether the observed reduction in writhing indicates true analgesia, sedation‐induced immobility, or nonspecific effects on baseline activity.

$$\text{Percent effect}=\frac{\mathrm{No}\;\text{of writhes in Control}-\text{Number of writhes in test}}{\mathrm{No}\;\text{of writhes in Control}\;}\times 100$$

Dose selection for the crude extract (25–100 mg/kg) and IONPs (2.5–7.5 mg/kg) was reported by previous pharmacological studies and preliminary experimental observations (Bukhari et al. 2016; Rauf, Ahmad, et al. 2024). Lower doses were selected for the IONPs because nanoparticle‐based formulations have been reported to enhance the pharmacological performance of bioactive compounds through improved surface area, dispersion, and interaction with biological systems (Ahmad et al. 2024). Therefore, the IONP doses were evaluated within a lower concentration range to determine whether comparable biological activity could be achieved.

#### 2.8.2 Sedative Activities (Open Field Test [OFT])

The synthesized nanoparticles' sedative activity and crude extract were evaluated using the OFT. The apparatus consisted of a square box (50 × 50 × 30 cm) with a white floor divided into 25 equal squares (10 × 10 cm) by black markers, placed in a soundproof room with controlled lighting. Animals in this experiment were divided into groups (*n* = 8 per group) and a negative control (normal saline, 10 mL/kg, IP), a positive control (diazepam, 0.5 mg/kg, IP), and test groups receiving varying doses of the extract (25, 50, and 100 mg/kg, IP) or iron nanoparticles (2.5, 5, and 7.5 mg/kg, IP). To maintain pharmacological consistency, the dose ranges used for analgesic evaluation were also employed here. The reference dose for diazepam (0.5 mg/kg) followed previously established OFT protocols (Mamun‐Or‐Rashid et al. 2017), while the extract doses were based on previously reported literature that shows sedative effects for *Eucalyptus* extracts (Shahwan et al. 2011). Nanoparticle doses were determined by previously reported toxicity assessments and studies indicating CNS effects at these concentrations (Singab et al. 2011). Locomotor activity was quantified by counting the number of squares crossed with all four paws; a reduction in crossings relative to the control group indicated sedative activity. The absence of an IONPs‐alone control in the analgesic assay limits the ability to distinguish between specific pain relief and nonspecific motor depression, such controls are required to account for baseline activity changes induced by the test compounds.

### 2.9 Molecular Docking

The molecular docking was conducted to observe the interaction of quercetin, which is a major component of *E. camaldulensis* extract. Quercetin (3,3′,4′,5,7‐pentahydroxyflavone) is a prominent flavonoid present abundantly in Eucalyptus species, including *E. camaldulensis* . Ghareeb et al. (2018) reported quercetin as one of the principal bioactive compounds in the methanolic extract of *E. camaldulensis* leaves through HPLC‐DAD analysis, confirming its quantitative significance. Ekinci et al. (2013) and Yang et al. (2025) reported that the presence of quercetin and related flavonoids is directly responsible for the reducing and capping capabilities of plant extracts in green nanoparticle synthesis, as these compounds possess abundant hydroxyl groups capable of donating electrons for metal ion reduction (Zhang and Elliott 2006). Molecular docking was conducted to examine the interaction of quercetin (a representative flavonoid previously reported in *E. camaldulensis* ; Chiamaka et al. 2019) and a simplified iron cluster model with the target enzymes: CA‐II, urease, XO, and cyclooxygenase‐2 (COX‐2). The docking simulations were performed to generate a hypothesis that shows insights into potential binding interactions, rather than to provide definitive mechanistic conclusions.

## 3 Results and Discussion

### 3.1 Phytochemical Results of *E. camaldulensis*

The result of the phytochemical screening test of methanol and ethanol leaf extracts of *E. camaldulensis* is given in Table 1. These results indicate that *E. camaldulensis* extracts, methanol and aqueous, comprise polar and nonpolar secondary metabolites, especially flavonoids, tannins, and saponins. These secondary metabolites have already been shown to exert high antioxidant and enzyme‐inhibitory activities. Flavonoids and phenolic compounds reported in *Eucalyptus* species may contribute to the reduction, stabilization, and capping of IONPs during green synthesis.

**TABLE 1:** Phytochemical screening *of Eucalyptus camaldulensis*.

Secondary metabolites	Hexane	Ethyl acetate	Methanol	Aqueous
Alkaloids	−	+	+	+
Flavonoids	−	+	+	+
Carbohydrates	−	+	+	+
Saponins	−	+	+	+
Tannins	−	+	+	+
Anthraquinones	−	−	−	−

### 3.2 Characterization

The stock solution is mixed with the solution containing iron chloride salt. The color change from yellow to black signified the formation of nanoparticles (Kumar et al. 2023). Functional groups were identified using FTIR analysis, and the formation of nanoparticles was confirmed using UV–visible spectroscopy (Salem and Fouda 2021; Sathya et al. 2018).

#### 3.2.1 UV–Visible Spectroscopy

**FIGURE 1:**
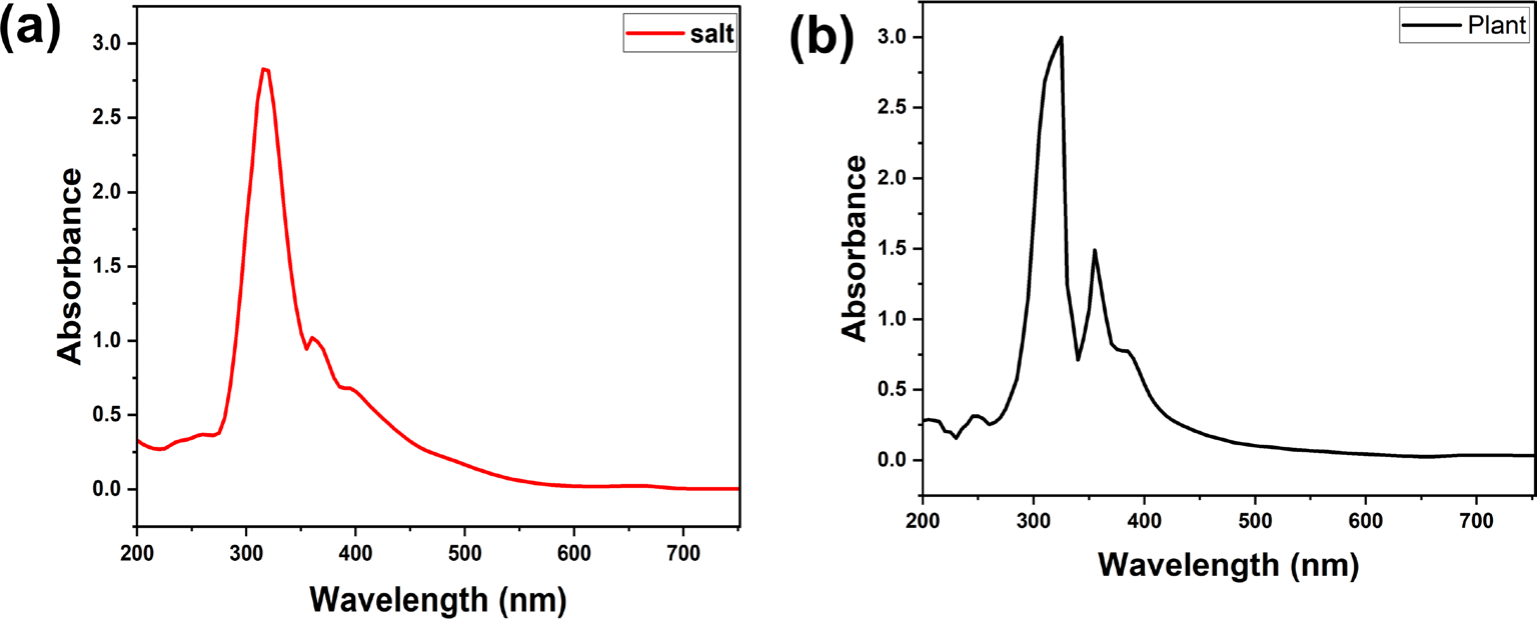
UV–Visible absorption spectra of (a) FeCl_3_·6H_2_O and (b) *Eucalyptus camaldulensis* extract after IONP synthesis. Absorption peaks at 272 and 310 nm confirm nanoparticle formation. Representative spectra from three independent syntheses.

UV–visible spectroscopy is the first indication in the formation of nanoparticles, and we also know that most of the nanoparticles give λmax in the range of 280–550 nm (Kumar et al. 2023). In Figure 1a, *λ*_max_ is given in the range of 272 nm, and in Figure 1b, *λ*_max_ is given in the range of 310 nm, which indicates the formation of nanoparticles in plant extract. IONPs often exhibit absorption in the range of 250–350 nm due to the charge transfer and electronic transition, so it is suggested that the Iron nanoparticles formed are in the form of Iron oxide rather than pure metallic Iron (Marslin et al. 2018).

#### 3.2.2 FTIR

**FIGURE 2:**
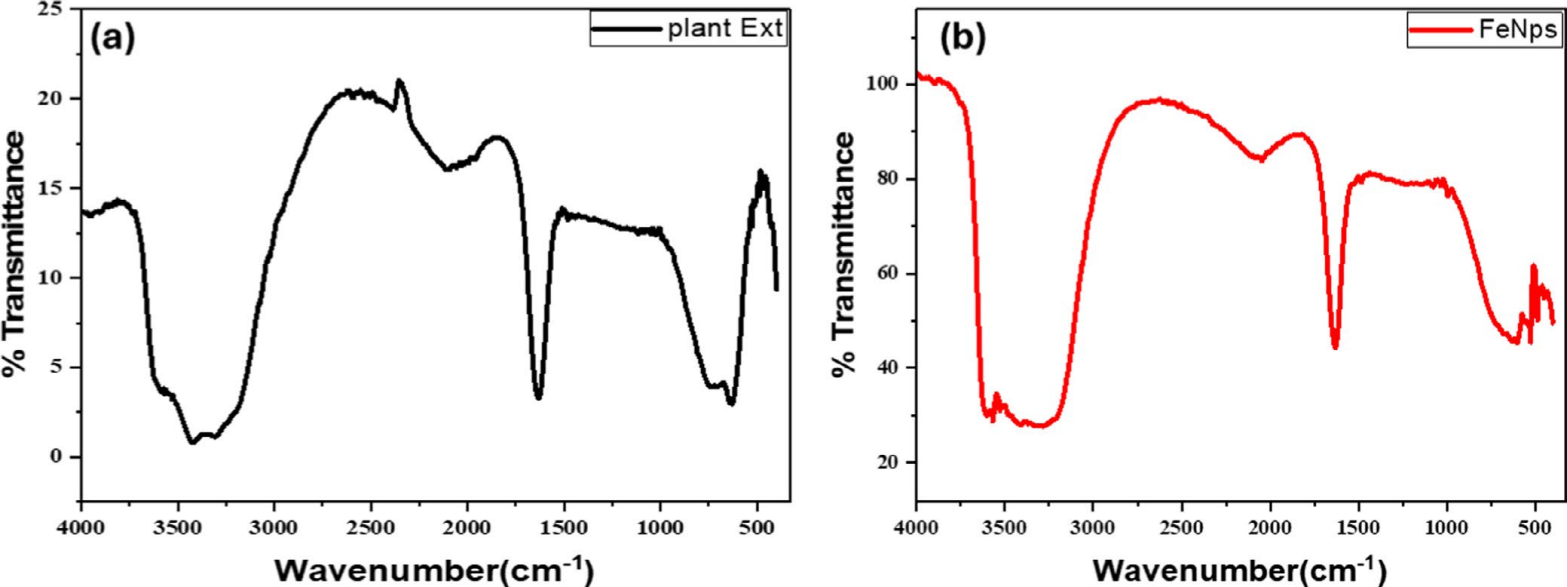
FTIR spectra of (a) *Eucalyptus camaldulensis* extract and (b) IONPs. Peaks at 3000–3500 cm^−1^ (O–H), ~1600 cm^−1^ (C═O), and 500–800 cm^−1^ (Fe–O) indicate functional groups involved in reduction and stabilization. Representative spectra from three independent measurements.

The FTIR spectrum of IONPs provides valuable information about the functional groups and bonds present in the sample. The broad peak observed in Figure 2 around 3000–3500 cm^−1^ corresponds to the O–H stretching vibrations, which indicate the presence of hydroxyl groups or adsorbed water molecules on the nanoparticle surface. This suggests some degree of surface hydration or stabilization. The prominent band at approximately ~1600 cm^−1^ corresponds to C═O stretching vibrations, which are associated with either carboxyl groups or organic molecules that served as capping/stabilizing agents during the reactions. The bands in the area before 1000 cm^−1^, particularly, those in the range of 500–800 cm^−1^, indicated there were Fe–O bonds associated with indicated iron oxide (Fe–O) formation in the nanoparticles. The presence of these functional groups and associated bonds will no doubt affect FeNP stability, surface reactivity, surface properties, and potential applications.

#### 3.2.3 SEM

**FIGURE 3:**
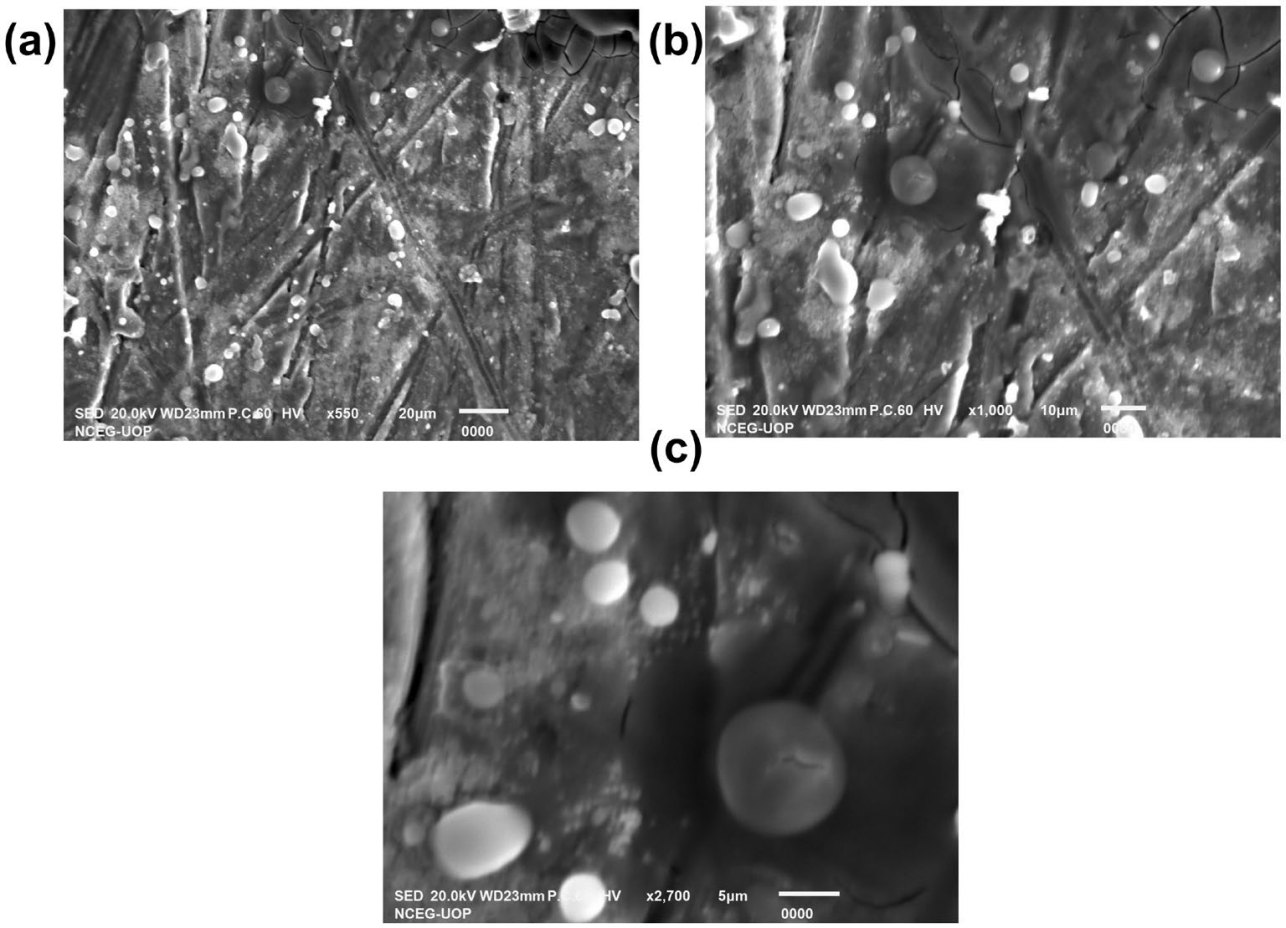
SEM images of IONPs at (a) ×550 and (b) ×2700 magnification showing surface morphology, particle distribution, and aggregation. Samples were sputter‐coated with Pt‐Au prior to imaging. Representative images from three independently synthesized batches. Scale bars: 50 μm (a) and 10 μm (c).

The surface morphology, size, and structural characteristics of the synthesized nanoparticles were checked using SEM. SEM analysis of IONPs synthesized with *E. camaldulensis* extract provides a complete picture of the IONPs (and their distribution). At the magnification of ×2700 (shown in Figure 3). The IONPs also had some aggregation, based on their irregular shapes, likely due to the use of organic compounds from the extract. With higher magnification, some surface roughness, as well as clusters on smaller scales, was observed. Once again, the nanoscale characteristics of each of the particles were illustrated through SEM techniques. At a lower magnification of ×550, the IONPs are distributed over a broader surface, showcasing a more macroscopic view of their dispersion. The particles appear heterogeneously scattered, with a mix of isolated particles and clusters, while the textured background suggests the influence of organic residues or substrate material (Figure 3).

### 3.3 In Vitro Enzyme Inhibition Activities

#### 3.3.1 Urease Inhibition

The extract and Iron nanoparticles were evaluated for urease antagonistic effect as shown in Table 2. The results show that extract exhibited 49.43% inhibition. In contrast, the IONPs showed enhanced activity with 95.49% inhibition and a lower IC_50_ value of 4.11 ± 1.32 μg/mL. The standard inhibitor thiourea demonstrated the highest activity with 97.01% inhibition and an IC_50_ of 0.22 ± 1.00 μM, indicating superior potency compared to both tested samples.

**TABLE 2:** Urease inhibitory activity of the extract and the synthesized IONPs from *Eucalyptus camaldulensis* .

Sample	Concentration	% Inhibition	IC_50_
Extract	0.2 μg/mL	49.43	—
IONPs	0.2 μg/mL	95.49	4.11 ± 1.32 μg/mL
Thiourea	0.2 μM	97.07	0.22 ± 1.00 μM

#### 3.3.2 Effect on CA‐II

Both of the tested samples demonstrated a variable effect against CA‐II, as shown in Table 3. A significant inhibitory activity was noted against IONPs with a 72.23% effect. While the extract does not antagonize the CA‐II at a significant level. The positive control drug was used as acetazolamide (82.29%).

**TABLE 3:** Carbonic anhydrase II assay of the extract and the synthesized IONPs from *Eucalyptus camaldulensis* .

Sample	Concentration (μg/mL)	% Inhibition	IC_50_
Extract	0.2 μg/mL	27.65	—
IONPs	0.2 μg/mL	72.23	28.98 ± 1.62 μg/mL
Acetazolamide	0.2 μM	82.29	16.42 ± 1.09 μM

#### 3.3.3 Effect on XO

In this case, both of the tested samples significantly antagonized the selected enzyme as presented in Table 4. The positive control drug (Allopurinol) showed the most significant effect with 96.07% effect. The percent inhibitory effect of the extract and IONPs was 70.12% and 86.03%, respectively.

**TABLE 4:** Effect on XO of the extract and the synthesized IONPs from *Eucalyptus camaldulensis* .

Sample	Concentration (μg/mL)	% Inhibition	IC_50_
Extract	0.2 μg/mL	70.12	18.78 ± 1.65 μg/mL
IONPs	0.2 μg/mL	86.03	7.03 ± 1.10 μg/mL
Allopurinol	0.2 μM	96.07	0.70 ± 0.87 μM

### 3.4 Analgesic Effect

A dose‐dependent analgesic effect was noted against both of the tested samples, as shown in Table 5. The analgesic effect of IONPs was more significant than the extract. The maximum percent effect of the extract at a higher dose (100 mg/kg) was 57.23%. The analgesic effect of IONPs was significant at lower (2.5 mg) and higher doses (7.5 mg), with a percent effect of 69.21 and 85.32, respectively.

**TABLE 5:** Analgesic activity of the extract and the synthesized IONPs from *Eucalyptus camaldulensis* .

Treatment	Dose (mg/kg)	Percent effect
Saline	10 mL/kg	—
Extract	25	41.41 ± 1.09**
	50	52.66 ± 1.18**
	100	57.23 ± 1.14***
IONPs	2.5	69.21 ± 1.08***
	5.0	78.43 ± 1.04***
	7.5	85.32 ± 1.00***
Diclofenac sodium	5	86.32 ± 0.40***

*Note:* Percent effect = [(writhes_control − writhes_test)/writhes_control] × 100. Statistical significance was determined using one‐way ANOVA with Tukey's post hoc test. An IONPs‐alone control group (without acetic acid challenge) was not included; therefore, reduced writhing may reflect sedation or nonspecific motor depression rather than true analgesia.

**p < 0.01, ***p < 0.001 compared to saline control group.

### 3.5 Sedative Effect

Sedative effects in a dose‐dependent manner, were exhibited by both tested samples as shown in Table 6. The crude extract demonstrated a considerable reduction in locomotor activity at the highest tested dose (100 mg/kg), while IONPs produced a dose‐dependent sedative effect across all tested doses (2.5, 5.0, and 7.5 mg/kg). Statistical significance was also determined using one‐way analysis of variance (ANOVA) followed by Tukey's post hoc test, with significance thresholds set at *p < 0.05, **p < 0.01, and ***p < 0.001.

**TABLE 6:** Sedative effect of crude extract and the synthesized IONPs from *Eucalyptus camaldulensis* .

Treatment	Dose (mg/kg)	Number of lines crossed (10 min)	% reduction
Saline	10 mL/kg	166.65 ± 0.61	—
Extract	25	60.88 ± 2.34***	63.5
	50	52.09 ± 2.04***	68.7
	100	44.21 ± 1.83***	73.5
IONPs	2.5	26.99 ± 1.65***	83.8
	5.0	17.34 ± 1.66***	89.6
	7.5	10.65 ± 1.07***	93.6
Diazepam	0.5	1.14 ± 1.37***	99.3

*Note:* Data are presented as mean ± SEM (*n* = 8 animals per group). Statistical significance was determined using one‐way ANOVA with Tukey's post hoc test.

***p < 0.001 compared to saline control group.

### 3.6 Statistical Analysis

All experiments were perfomed in triplicate, and the data were expressed as mean ± standard deviation (SD) unless otherwise noted. For the in vitro enzyme inhibition assays especifically targeting urease, CA II, and XO of which each concentration was tested in three independent experiments (*n* = 3), with percent inhibition values calculated relative to the control wells. In the in vivo studies, which evaluated analgesic and sedative activities, groups consisted of eight animals each (*n* = 8), and results were presented as mean ± standard error of the mean (SEM). Before statistical analysis, the normality of the data distribution was verified using the Shapiro–Wilk test. Significant differences between the multiple groups were determined by using statistical analysis (ANOVA) followed by Tukey's post hoc test for pairwise comparisons. Comparisons between two specific groups were performed using an unpaired, two‐tailed Student's *t*‐test. For comparisons between two groups, Student's *t*‐test (two‐tailed, unpaired) was applied. A significance threshold of *p* < 0.05 was considered statistically significant, with *p* < 0.01 and *p* < 0.001 indicating moderate and highly significant differences, respectively. Outliers were identified using Grubbs' test (*α* = 0.05) and were excluded from analysis only if a technical error was confirmed; otherwise, all data points were retained. No data normalization was applied as all assays were internally controlled. IC_50_ values for enzyme inhibition were calculated by nonlinear regression analysis using a four‐parameter logistic curve fit (variable slope) in GraphPad Prism (version 8.0, GraphPad Software, San Diego, CA, USA). All other statistical analyses were performed using SPSS (version 25.0, IBM Corp., Armonk, NY, USA). Enzyme inhibition assays (in vitro) (urease, CA II, and XO), each concentration was tested in triplicate (*n* = 3 independent experiments), and percent inhibition values were calculated relative to control wells. For in vivo studies (analgesic and sedative activities), eight animals were used per group (*n* = 8), and the results were expressed as mean ± SEM. Comparisons between multiple groups were also performed using one‐way ANOVA followed by Tukey's post hoc test for pairwise comparisons. For comparisons between two groups, Student's *t*‐test (two‐tailed, unpaired) was applied. For comparisons between two groups, Student's *t*‐test (two‐tailed, unpaired) was applied. A significance threshold of *p* < 0.05 was considered statistically significant, with *p* < 0.01 and *p* < 0.001 indicating moderate and highly significant differences, respectively. Outliers were identified using Grubbs' test (*α* = 0.05) and were excluded from analysis only if a technical error was confirmed; otherwise, all data points were retained. No data normalization was applied as all assays were internally controlled. IC_50_ values for enzyme inhibition were calculated by nonlinear regression analysis using a four‐parameter logistic curve fit (variable slope) in GraphPad Prism (version 8.0, GraphPad Software, San Diego, CA, USA). All other statistical analyses were performed using SPSS (version 25.0, IBM Corp., Armonk, NY, USA).

### 3.7 Molecular Docking

#### 3.7.1 Quercetin Interactions With Selected Enzymes

**FIGURE 4:**
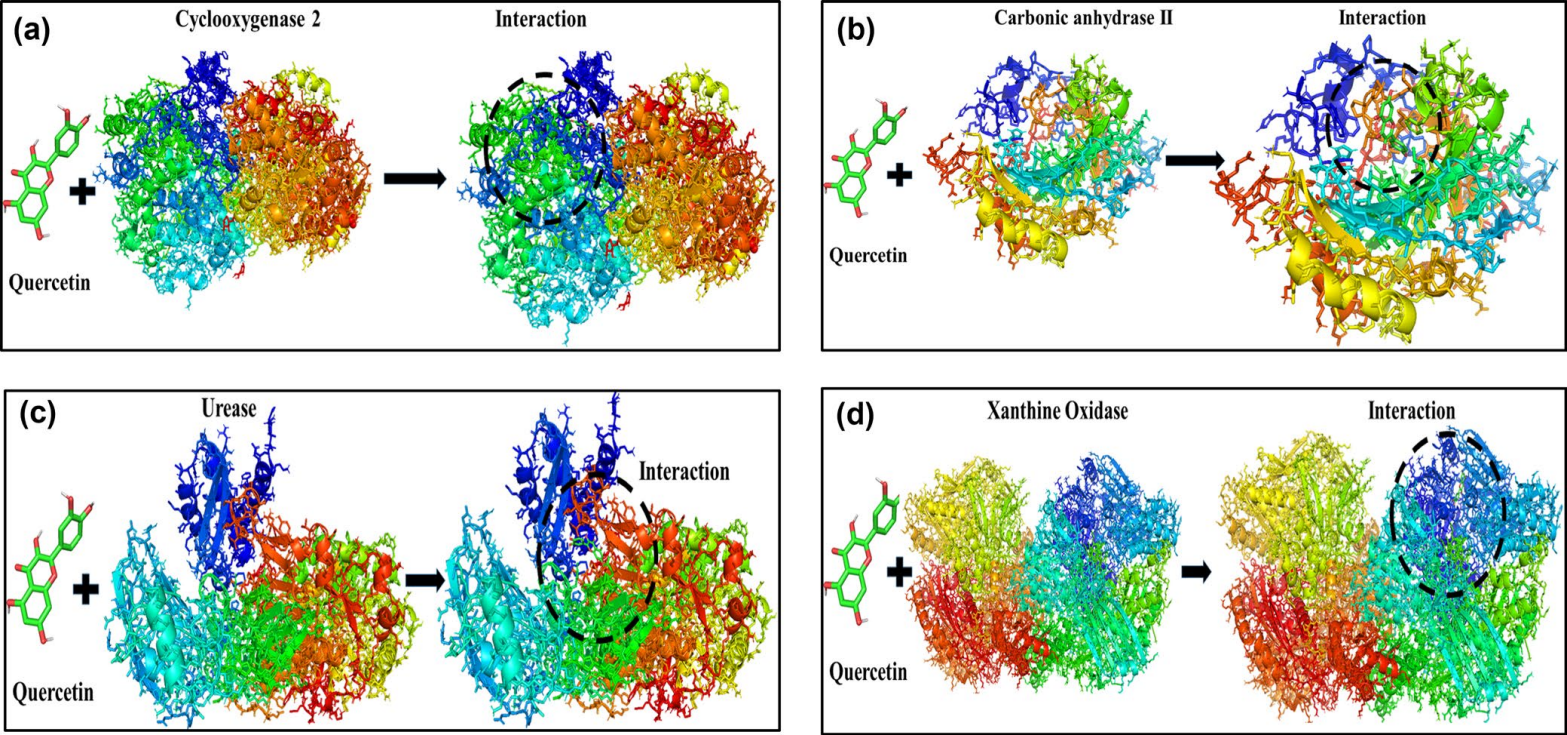
Predicted binding poses of quercetin with (a) COX‐2, (b) CA‐II, (c) urease, and (d) XO. Quercetin (green sticks) interacts with key residues (light blue) via hydrogen bonds (yellow dashes) and metal coordination (gray dashes). Binding energies (kcal/mol): COX‐2: –8.8, CA‐II: −7.6, urease: –8.9, XO: −9.5. Best poses from 10 independent docking runs.

To validate further the experimentally observed results, molecular docking simulations were performed that gave insightful details of the quercetin and DFT‐optimized IONPs' binding interactions with the targeted enzymes, including CA II, urease, and XO as shown in Figure 4. The crystallographic information file (CIF) of Fe, for the nanoparticle, was obtained from the Materials Project (an open database). The unit cell was optimized via density functional theory (DFT) and calculations were implemented via the SIESTA code. Prior to interaction, the targeted enzymes obtained from the Protein Data Bank (PDB) (chosen based on high resolution and relevance to previously reported studies) went through several processes, including removal of co‐crystallized ligands, solvent molecules, and nonessential ions. Polar hydrogen atoms were added, and appropriate partial charges (Kollman charges) were applied to avoid any pseudo interactions. Finally, energy minimization of the enzyme was carried out using the AMBER force field (ff14SB) to adjust atomic coordinates to remove stereochemical clashes, improving geometry, and achieve a stable (low‐potential energy state). However, the most important aspect to note is that the crystallographic enzyme structures used for docking may not exactly correspond to the enzyme sources and can be considered a limitation of the present study.

The docking analysis of quercetin divulged a very strong and satisfactory binding affinity toward the catalytic site of COX‐2, as shown in Figure 4a, with a minimum binding energy of −8.8 kcal/mol. This molecule networks with important residues like Tyr385 and Trp387; quercetin's aromatic core is involved in *π*–*π* stacking, which is considered to be a hallmark interaction for many known COX‐2 inhibitors. While the hydroxyl substituents of quercetin's are involved in hydrogen bonding with Tyr355, Arg120, and Ser530, securing the ligand in a way that directly competes with arachidonic acid, the natural substrate. Through COX‐2 competitive inhibition, these connections propose structural support for quercetin's analgesic and anti‐inflammatory properties.

In molecular docking, the Quercetin also showed strong binding with CA‐II with a score of −7.6 kcal/mol (Figure 4b). The mode of binding was prominent for direct coordination with the catalytic Zn^2+^ ion, typical of traditional inhibitors of the sulfonamide type. The coordination with metal was augmented with hydrogen bonding with residues Thr199 and Glu106, while hydrophobic stabilization was via Val121, Phe131, and Leu198. The combination of the interactions implies that quercetin may function as a competitive inhibitor of the hydration of carbon dioxide at the site of the enzyme in the cytosol and may therefore warrant further investigation in relation to CA‐II‐associated biological pathways.

Quercetin in comparison with *Bacillus pasteurii* urease, the binding of quercetin was highly favorable (−8.9 kcal/mol), in agreement with reported antiulcer and antiurease activities (Figure 4c). The predicted binding conformation indicated that quercetin entered the nickel‐bearing active site and formed coordination bonds with the Ni1 and Ni2 cations, an essential prerequisite for effective urease activity inhibition. H‐bonding with Hisα222, Alaα170, and Aspα223, as well as hydrophobic contacts with Metα366 and Hisα275, substantiated the complex stability. The tenancy of the binding cavity with quercetin is predicted to prevent the access of the endogenous urea substrate, consequently decreasing enzymatic turnover. The metal ion coordination, hydrogen bonding, and hydrophobic stabilization provide cumulative and a solid explanation for the strong anti‐urease activity in biological assays. The interaction of quercetin found with XO was most effective (Figure 4d) with a docking score of −9.5 kcal/mol. Several hydrogen bonds with Glu802, Arg880, and Thr1010, along with extensive *π*–*π* stacking with Phe914 and Phe1009, provided high quercetin–XO complex stability. In purine catabolism as well as in the formation of uric acid, it exists as a prime enzyme; the findings constitute strong evidence favoring the therapeutic role of quercetin as a potent candidate against gout with the potential of alleviating hyperuricemia. Unlike quercetin, which largely worked through competitive occupation of the active sites, the Fe nanoparticles showed an independent mode of inhibition with strong surface binding, steric blockage, and allosteric modulation. The Fe nanoparticles exhibited strong binding with COX‐2 with a docking score of −8.6 kcal/mol. The nanoparticles interacted with residues at or near the entrance of the catalytic channel, including Arg120, Lys83, and His90, through electrostatic forces, while supplementary stabilization was provided through hydrophobic contacts with Tyr115, Phe318, and Trp387. The pose cluster with essentially the same binding energies indicated the presence of a favored, highly stable binding site. The associated mode of interaction suggests a non‐competitive mechanism in that IONPs could sterically hinder substrate access or create conformation changes that reduce enzymatic function.

#### 3.7.2 IONPs Interaction With Selected Enzymes

**FIGURE 5:**
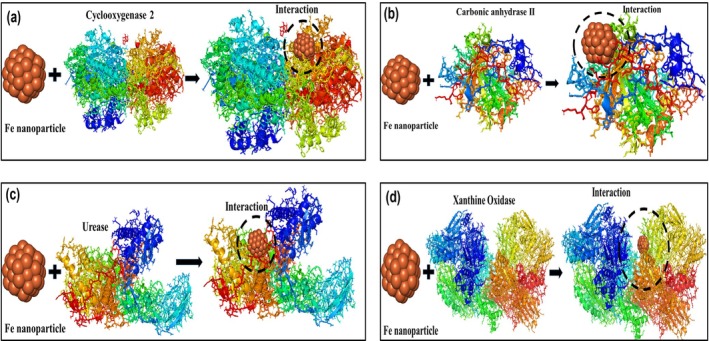
Predicted binding poses of Fe nanoparticles with (a) COX‐2, (b) CA‐II, (c) urease, and (d) XO. Fe NPs (spheres) interact with key residues (light blue) via electrostatic interactions (purple dashes) and hydrophobic contacts (gray surfaces). Binding energies (kcal/mol): COX‐2: –8.6, CA‐II: −9.8, urease: –9.9, XO: −10.6. Best poses from 10 independent docking runs.

Similarly, the IONPs showed a strong binding affinity of −9.8 kcal/mol in the case of the CA‐II, with low RMSD values in all top poses (Figure 5). This suggests a stable and specific mode of interaction. Strong electrostatic interactions with Arg67, Lys91, and Lys170 provided additional anchoring, and the nanoparticles were observed to coordinate with proton‐shuttling residues such as His64. With Val121 and Phe131, hydrophobic contacts also consolidated the complex, and this would indicate that IONPs could interrupt the orientation of catalytic residues and proton shifting critically in enzymatic function. This mechanism seems non‐competitive in as much as the active Zn^2+^ ion is not directly involved, but instead, access is blocked and active site geometry is distorted. Fe nanoparticles against urease demonstrated the strongest interactions, with an unusually high binding energy of −9.9 kcal/mol. The docking study disclosed direct coordination with nickel‐binding residues Hisα222 and Hisα275, mimicking the ligands responsible for stabilizing the catalytic Ni cluster. The latter was supported by electrostatic interaction with Lysα220 and Argα339 and hydrophobic stabilization with Alaα170 and Metα366. The prophesied mode of action was dual inhibition, both through sterically inhibiting substrate access and through destabilizing the coordination environment of the nickel ions. This type of mechanism would be particularly effective in inhibiting urease activity, consequently capping ammonia production and urease‐based pathogenicity. Fe nanoparticles consequently showed the strongest total interaction with XO, with a docking value of −10.6 kcal/mol, better than even that of quercetin. The nanoparticles interacted with critical residues within the molybdenum‐pterin (Moco) region, namely Cys992 and Cys1014, that oversee coordinating the molybdenum. Through Glu802 and Arg880, stabilization via electrostatic means was provided, while *π*–*π*‐cation and hydrophobic contacts with Phe798, Phe914, and Phe1009 further solidified the stability of the complex. The docking results suggest that Fe nanoparticles may occupy the tunnel‐shaped pore leading to the catalytic center, potentially acting as a steric barrier that could limit substrate access. This mode of interaction, if confirmed experimentally, may offer an alternative approach for XO inhibition with potential relevance to hyperuricemia management.

The docking simulations uncover two mutual modes of inhibition: quercetin elicits competitive inhibition through the occupation of active sites with strong hydrogen bonding, *π*–π stacking, and metal coordination, whereas Fe nanoparticles act through non‐competitive pathways that involve steric blockage, allosteric modulation, and interruption of central metal coordination networks. The relatively low‐temperature binding energies systematically extracted in both the quercetin (−7.6 to −9.5 kcal/mol) and the Fe nanoparticle (−8.6 to −10.6 kcal/mol) complexes provide robust computational evidence in support of their potential role as multifunctional inhibitors, as illustrated in Figure 5. These findings provide preliminary support for further investigation of flavonoid‐associated and nanoparticle‐mediated interactions with enzyme targets relevant to inflammation, microbial urease activity, gastric disorders, and gout‐related pathways.

## 4 Discussion

Medicinal plants are traditional therapeutic agents. In the presynthetic drug era, human beings globally depended on herbs as medicines. The negative side effects and lack of compliance with synthetic drugs have revived the interest in plant‐based remedies in the present times. *E. camaldulensis* was used traditionally for rheumatism and neuropathic pain, but evidence of mechanisms was insufficient. Using in vitro, in vivo, and molecular docking methods, the current study elucidated the activity of *E. camaldulensis* extract and IONPs.

The IONPs demonstrated a very efficient urease inhibition rate of 95.49%. This high level of activity suggests they could be effective against pathogens like *Helicobacter pylori* , which depends on the urease enzyme to neutralize stomach acid and ensure its survival (Bhuiyan et al. 2020). These results support the existing research indicating that green‐synthesized nanoparticles can effectively disrupt urease function, thereby undermining bacterial stability in acidic conditions. In line with this, the significant suppression of CA II (72.23%) suggests the potential use of the compound as an antihypertensive and antiglaucoma. To inhibit the catalytic activity of the zinc ion or the proton‐shuttling residues of CA‐II enzyme activity, plant‐capped nanoparticles were reported, without direct coordination to the zinc center. This noncompetitive binding site, which our docking simulations validate, could be more selective and have fewer side effects than conventional sulfonamide inhibitors.

The inhibition of XO was high (86.03%) and is important, as XO plays a critical role in purine metabolism and in the synthesis of uric acid, a key target in the gout management. The Docking simulations suggested special binding sites of IONPs to the molybdenum‐pterin (Moco) site on XO, involving a steric‐blocking mechanism that may differ from conventional small‐molecule inhibitors such as allopurinol (Berman et al. 2019). Further kinetic and structural studies are needed to confirm this mechanism. XO‐inhibition corresponds to the folkloric use of the plant in joint pains and rheumatism.

The in vivo and multimodal pharmacology study of these IONPs is further confirmed by the presence of analgesic and sedative effects. IONPs can reduce XO activity concomitant with a possible effect on COX‐2 and central pain pathways, potentially resulting in synergistic analgesia. Other phytochemical‐loaded iron nanoparticles have been denoted to have analogous multimodal effects in which flavonoids and terpenoids combine with the metal core to traverse biological obstacles and target various targets (Huang et al. 2014).

These findings suggest that IONPs synthesized in this study using *E. camaldulensis* extract may function not only as carriers but will also act as active pharmacological agents, a dual functionality that distinguishes them from conventional inert nanocarriers. Studies are required to elucidate the in vivo mechanisms, optimize dosing regimens, and establish long‐term safety profiles before any therapeutic applications can be considered (Huber 2005).

While the docking simulations provided a very valuable mechanistic understanding about the binding of IONPs, certain methodological limitations should be acknowledged. The iron nanoparticle was modeled as a static DFT‐optimized structure, which does not fully capture the dynamic surface reorganization, the solvation effects, or protein flexibility that may occur in biological systems. Docking calculations assume a rigid protein conformation and do not account for the complex interplay of nanoparticle surface chemistry, protein corona formation, or potential multivalent binding interactions that may influence actual binding in physiological conditions. The coherent correlation between the docking predictions and experimental enzyme inhibition data supports the utility of these simulations as a paired tool for the mechanistic interpretation.

## 5 Conclusion

This study successfully reports the green synthesis of the IONPs using aqueous extract of leaves of *E. camaldulensis* . The NPs were characterized, confirming the NPs formation with characteristic absorption peaks at 272–310 nm, functional groups indicative of the phytochemical capping, and irregular spherical morphology with some aggregation. The IONPs exhibited potent enzyme inhibitory activity against the enzyme urease (95.49%), the CA II (72.23%), and XO (86.03%), with promising IC_50_ values. In vivo evaluations demonstrated dose‐dependent sedative properties during open‐field assays, alongside a significant attenuation of the nociceptive writhing response in acetic acid‐induced models at IONP dosages ranging from 2.5 to 7.5 mg/kg. However, due to the absence of an IONPs‐alone control group and the confounding sedative effects observed, the reduction in writhing could not be definitively attributed to a specific analgesic activity.

Molecular docking using quercetin (a representative flavonoid previously reported in *E. camaldulensis* ) and a simplified iron cluster model provided hypothesis‐generating insights that it has potential binding interactions with the target enzymes. Quercetin showed predicted binding affinities ranging from −7.6 to −9.5 kcal/mol, while the iron cluster model suggested potential electrostatic and hydrophobic contributions with predicted interaction energies from −8.6 to −10.6 kcal/mol. These computational findings were interpreted with caution, as the iron cluster model does not represent the full complexity of the synthesized IONPs (phytochemical coating, 20–50 nm size), and the urease docking was performed using a bacterial surrogate structure rather than jack bean urease used in vitro.

These findings collectively indicate that greenly synthesized IONPs using *E. camaldulensis* plant extract possess notable enzyme inhibitory and sedative properties that permit further investigation; the relative contributions of the IONP core versus the phytochemical coating to the observed activities remain unresolved. Investigation required for future should prioritize: (i) the quantitative profiling of surface‐bound phytochemicals via LC–MS or HPLC following standardized desorption protocols; (ii) a comprehensive physicochemical characterization utilizing XRD, TEM, DLS, and zeta potential analysis to ensure structural and colloidal stability; (iii) the pharmacological validation of analgesic efficacy through specialized assays, such as tail‐flick or hot‐plate tests, incorporating rigorous control cohorts; (iv) the elucidation of molecular mechanisms through antagonist challenges and receptor‐binding studies; (v) the assessment of chronic toxicity and pharmacokinetic (ADME) profiles; and (vi) the experimental validation of predicted molecular interactions using high‐sensitivity biophysical techniques, such as surface plasmon resonance (SPR) or isothermal titration calorimetry (ITC). Such multidimensional analyses are essential to substantiate the therapeutic viability of these IONPs for the management of pain and enzyme‐associated pathologies.

## 6 Limitations of the Study

Several methodological constraints merit consideration within this study. The Phytochemical analysis was limited to qualitative screening, which confirm the presence of major bioactive compounds classes but does not provide compound‐specific identification or quantification; while we selected quercetin as a representative ligand based on extensive literature (Ekinci et al. 2013; Ghareeb et al. 2018). Its presence in the specific extract that was used was not experimentally confirmed. In vivo studies using animal's model were conducted using a single rodent species without sex‐based subgroup analysis or chronic toxicity assessment, and the dose selection was not informed by formal pharmacokinetics studies. Enzyme inhibition assays utilized purified enzymes rather than cell‐based systems, which may not fully sum up the biological microenvironment. In addition, the molecular docking simulations employed simplified static nanoparticle surface models along with rigid protein conformations, which may not accurately capture the dynamic nature of biological interactions under physiological conditions. Furthermore, the predicted binding interactions were not experimentally validated. Despite these limitations, the docking findings showed reasonable agreement with the experimental enzyme inhibition results, supporting their potential mechanistic relevance.

Thus, the combination of phytochemical screening, physicochemical characterization, in vitro enzyme inhibition assays, in vivo pharmacological evaluation, and computational modeling provides an integrated foundation for understanding the biological potential of the synthesized nanoparticles. However, further quantitative, mechanistic, and long‐term biological investigations are required to establish their therapeutic applicability more comprehensively.

## Author Contributions

**Shahkar Usama:** investigation, validation, formal analysis. **Arshad Iqbal:** conceptualization, investigation. **Abdulhakeem S. Alamri:** conceptualization, investigation. **Mohammed Mansour Quradha:** conceptualization, investigation, validation. **Walaa F. Alsanie:** conceptualization, methodology, software. **Jaseem Ahmad:** conceptualization, methodology, validation. **Zubair Ahmad:** conceptualization, investigation, validation. **Abdur Rauf:** conceptualization, investigation. **Naveed Muhammad:** conceptualization, investigation, validation. **Rahaf Ajaj:** conceptualization, investigation, validation. **Imran Ullah:** conceptualization, investigation, validation. **Zuneera Akram:** conceptualization, methodology, formal analysis.

## Conflicts of Interest

The authors declare no conflicts of interest.

## Data Availability

The data that support the findings of this study are available on request from the corresponding author. The data are not publicly available due to privacy or ethical restrictions.
